# Supplementary material for: Functional Study of Haemophilus ducreyi Cytolethal Distending Toxin Subunit B
Source: Toxins (Basel). 2020 Aug 19;12(9):530. doi: 10.3390/toxins12090530 (PMC7551728; doi:10.3390/toxins12090530)
Supplement: Supplementary file 1 [file toxins-12-00530-s001.pdf]

Supplementary Materials: Functional Study of *Haemophilus Ducreyi* Cytolethal Distending Toxin Subunit B.

Benoît J. Pons, Nicolas Loiseau, Saleha Hashim, Soraya Tadrist, Gladys Mirey and Julien Vignard

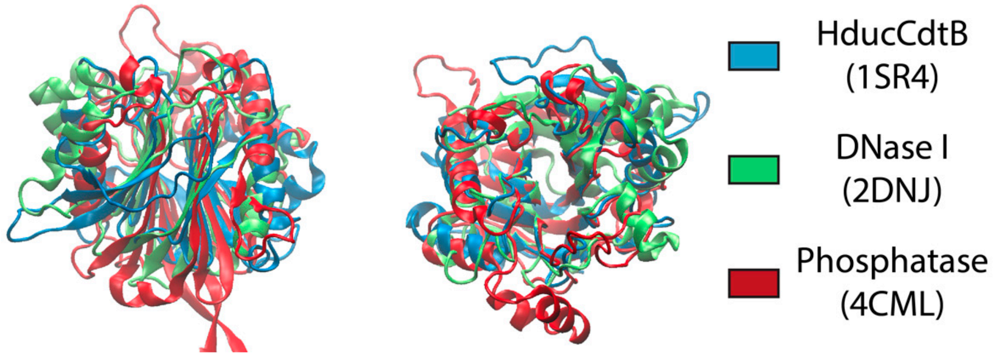

Figure S1. Structural alignment performed in this study as seen from the side (left panel) and the top (right panel). Superposition has been obtained after 3D-spatial alignment of the highly conserved catalytic residue asparagine (N273 of the HducCdtB) included in the typical super secondary beta-barrel structure. Colored Backbones of the protein structures correspond to HducCdtB (1SR4) in blue, Bovine DNase I (2DNJ) in green and Type II Inositol 1,4,5-trisphosphate 5- Phosphatase (4CML) in red.

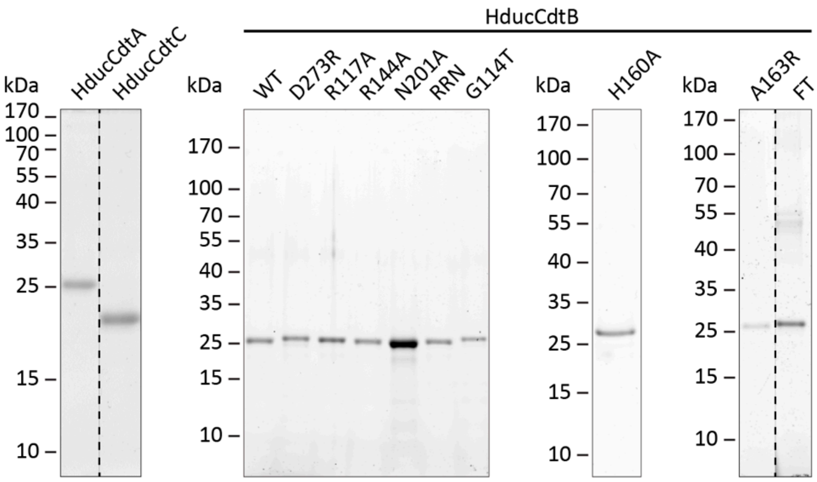

Figure S2. CDT subunits purification. SDS-PAGE analysis HducCdtA, HducCdtC and the HducCdtB mutant proteins purified from *E. coli* DH5α under denaturing conditions.

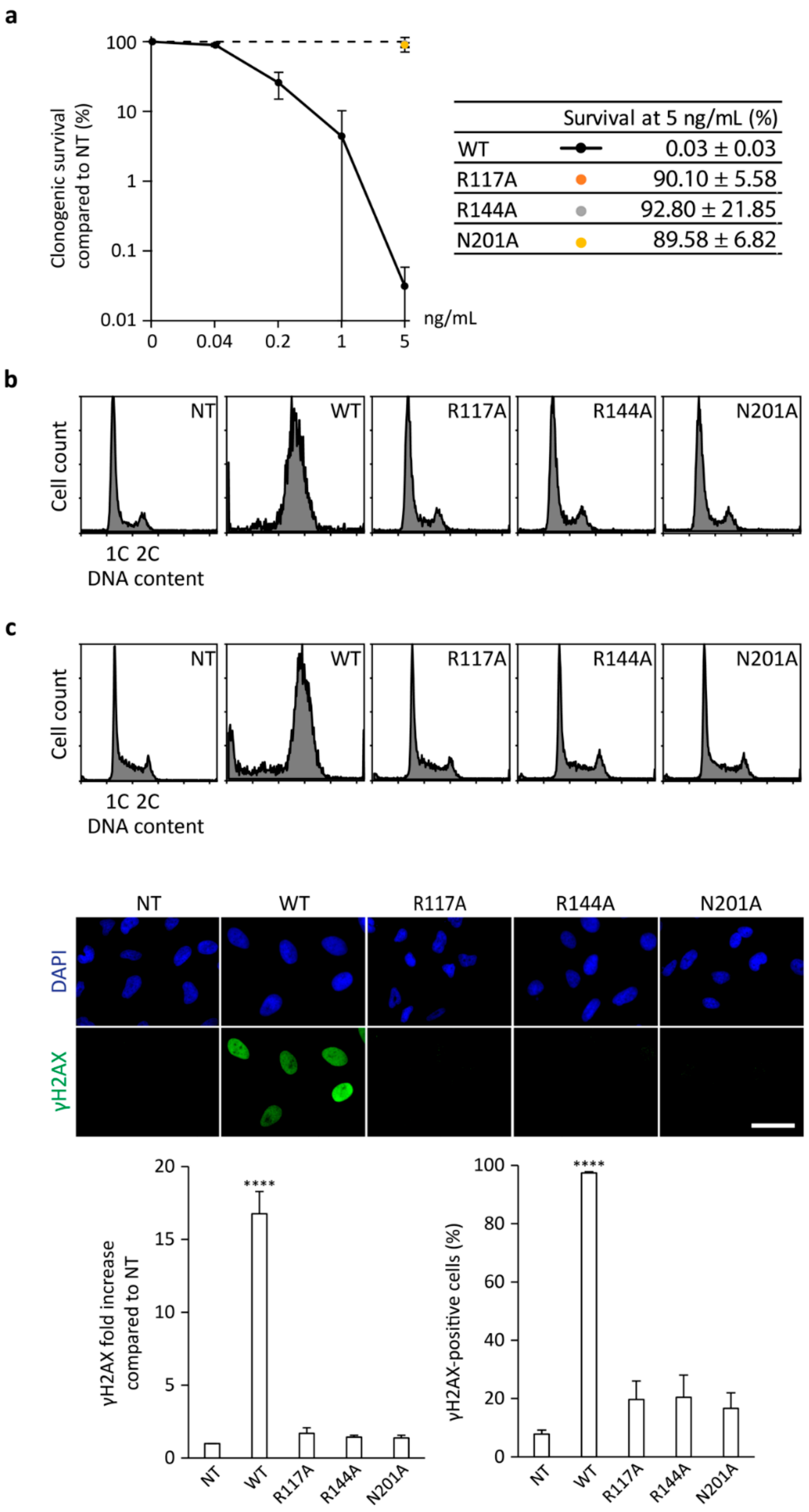
a
Clonogenic survival compared to NT (%)
100
10
1
0.1
0.01
0
0.04
0.2
1
5
ng/mL
Survival at 5 ng/mL (%)
WT 0.03 ± 0.03
R117A 90.10 ± 5.58
R144A 92.80 ± 21.85
N201A 89.58 ± 6.82
b
Cell count
NT
WT
R117A
R144A
N201A
1C 2C
DNA content
c
Cell count
NT
WT
R117A
R144A
N201A
1C 2C
DNA content
NT
WT
R117A
R144A
N201A
DAPI
γH2AX
γH2AX fold increase compared to NT
20
15
10
5
0
****
NT
WT
R117A
R144A
N201A
γH2AX-positive cells (%)
100
80
60
40
20
0
****
NT
WT
R117A
R144A
N201A

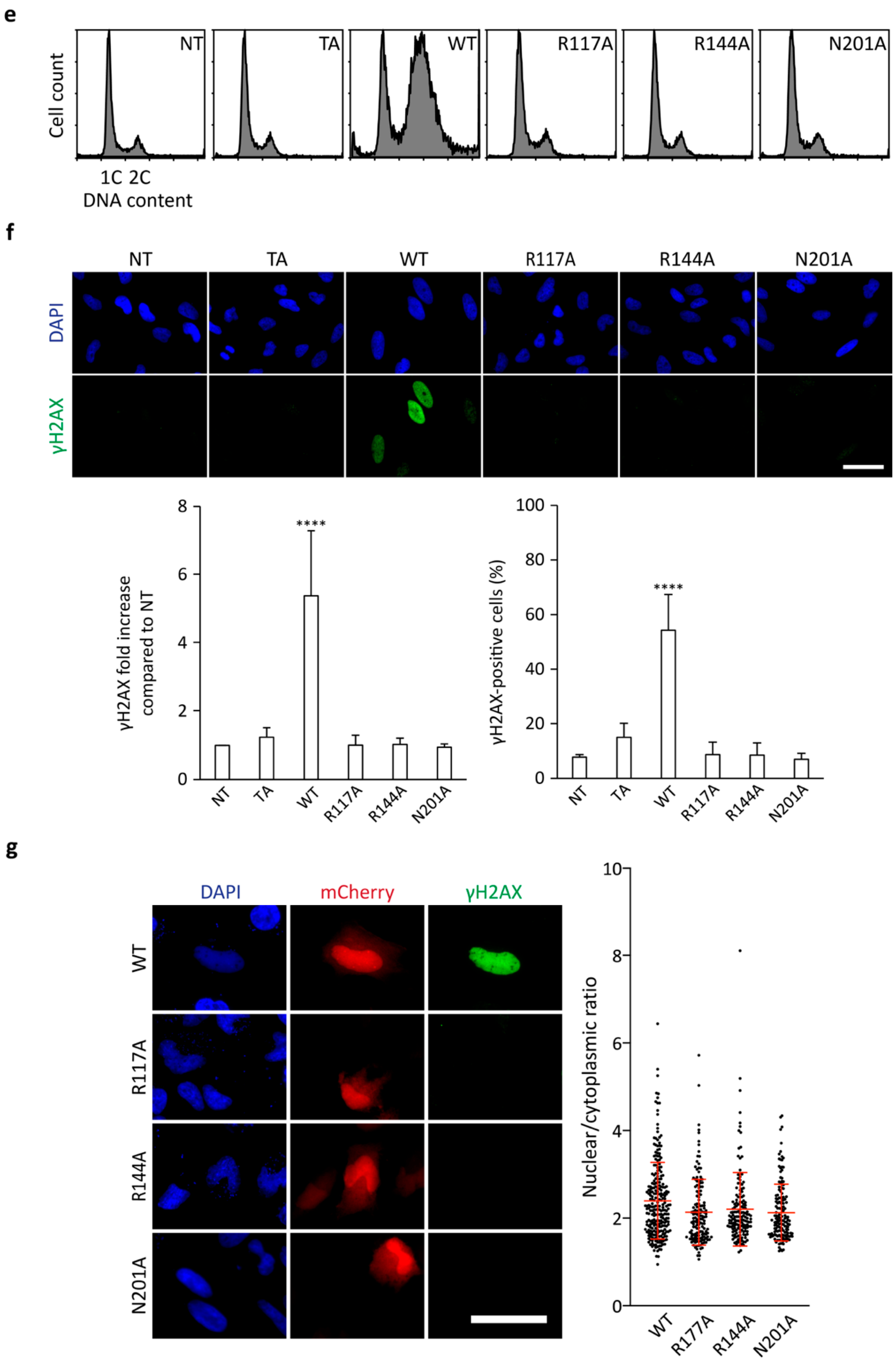
e
NT
TA
WT
R117A
R144A
N201A
Cell count
1C 2C
DNA content
f
NT
TA
WT
R117A
R144A
N201A
DAPI
γH2AX
γH2AX fold increase compared to NT
****
γH2AX-positive cells (%)
****
g
DAPI
mCherry
γH2AX
WT
R117A
R144A
N201A
Nuclear/cytoplasmic ratio
WT
R177A
R144A
N201A

Figure S3. Effects of DNA-binding single mutations on CDT and CdtB-induced cytotoxicity and DNA-damage. **(a)** Clonogenic survival of HeLa cells after 10 days of treatment with HducCDT mutants at the indicated concentrations. The table presents the mean survival at 5 ng/µL ± SD of three independent experiments. **(b)** Representative cell cycle analysis of HeLa cells after 36 h exposure to 35 ng/mL of HducCDT mutants. **(c)** Representative cell cycle analysis of Jurkat cells after 36 h exposure to 35 ng/mL of HducCDT. **(d)** Representative images of γH2AX immunofluorescence and associated γH2AX quantifications from HeLa cells treated with 35 ng/mL of HducCDT mutants for 24 h. Quantifications of γH2AX signal are represented as the mean fluorescence intensity per cell (normalized to 1 for the untreated condition) or as the proportion of γH2AX-positive cells. Results present the mean ± SD of at least three independent experiments. Statistical differences were analyzed between each CDT treatment and the untreated condition (****, p-value <0.0001). Scale bar: 50 µm. **(e)** Representative cell cycle analysis of HeLa cells transfected with 1 ng/mL of HducCdtB mutants for 36 h. **(f)** Representative images of γH2AX immunofluorescence and associated γH2AX quantifications from HeLa cells transfected with 1 µg/mL of HducCdtB mutants for 24 h. Quantifications of γH2AX signal are represented as the mean fluorescence intensity per cell (normalized to 1 for the untreated condition) or as the proportion of γH2AX-positive cells. Results present the mean ± SD of at least three independent experiments. Statistical differences were analyzed between each CDT treatment and the untreated condition (****, p-value <0.0001). Scale bar: 50 µm. **(g)** Representative images of γH2AX immunofluorescence, mCherry fluorescence and associated mCherry signal quantifications from HeLa cells transfected with pmCherry-C1-HducCdtB mutants for 14h. Quantifications are represented as the ratio for each individual cell of the nuclear mCherry signal over the cytoplasmic mCherry signal. Results present every cell analyzed over three independent experiments and the mean ± SD of these cells. Statistical differences were analyzed between CDT mutants and the WT CDT. Scale bar: 50 µm. NT: untreated cells, TA: transfecting agent.

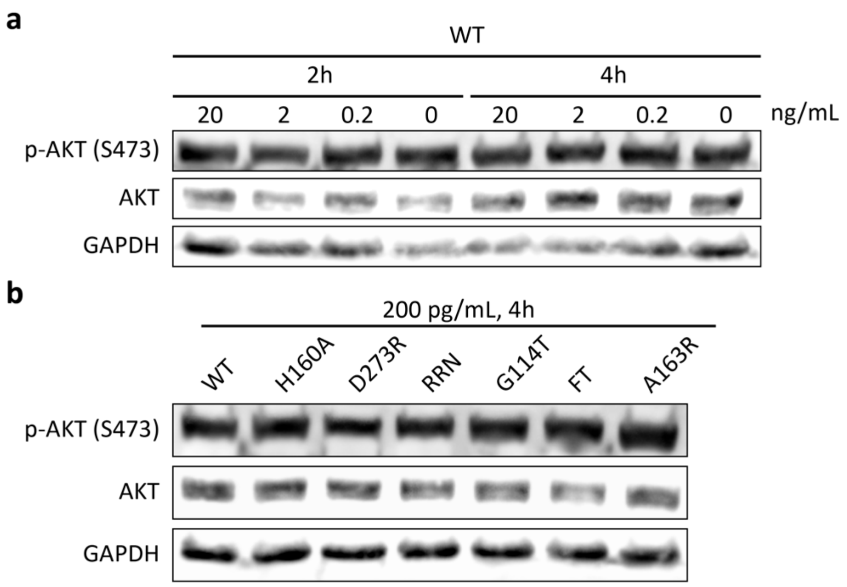

Figure S4. Absence of modulation of p-AKT after CDT exposure. **(a)** Jurkat cells were exposed to different concentrations of HducCDT WT for 2 or 4 hours and soluble extracts were prepared to assess the level of indicated proteins with specific antibodies by Western blot analyses. **(b)** Jurkat cells were exposed to 200 pg/ml of HducCDT WT or the indicated mutants for 4 hours and soluble extracts were prepared to assess the level of indicated proteins with specific antibodies by Western blot analyses.

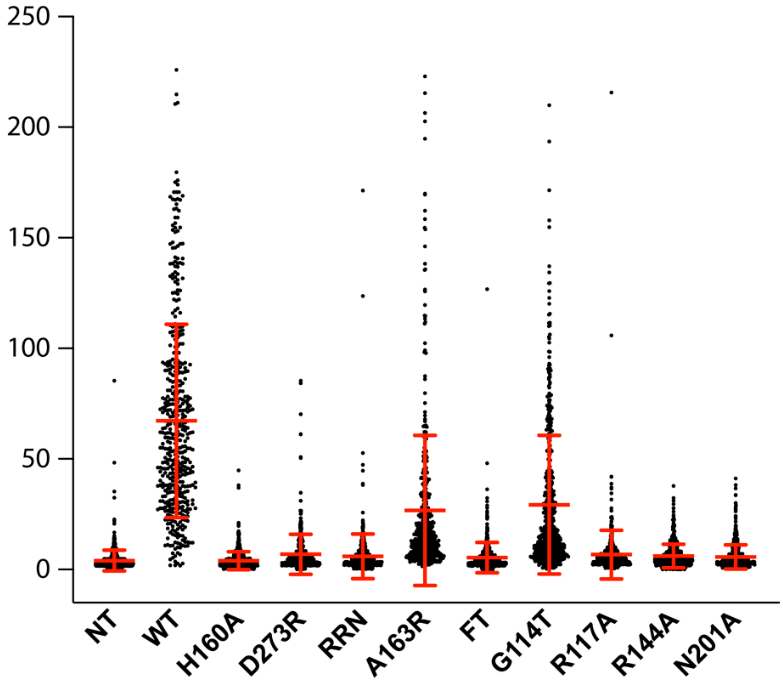

Figure S5. Single cell γH2AX signal quantifications of cells exposed to HducCDT mutants from Fig 4. Results present the raw fluorescence intensity of each cell from the different experiments, with the mean ± SD. NT: untreated cells.

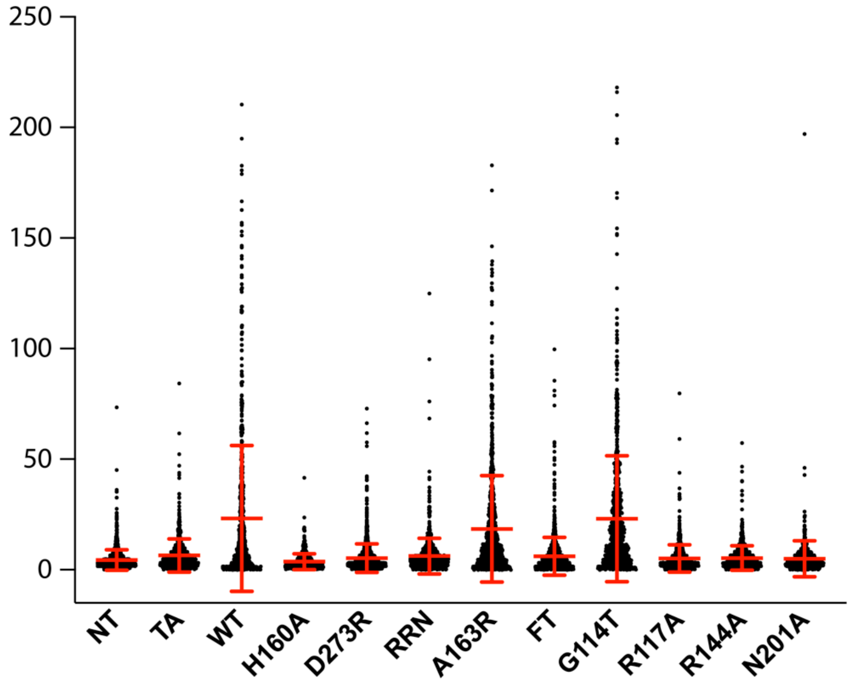

Figure S6. Single cell γH2AX signal quantifications transfected with HducCdtB mutants from Fig 5b. Results present the raw fluorescence intensity of each cell from the different experiments, with the mean ± SD. NT: untreated cells, TA: transfecting agent.

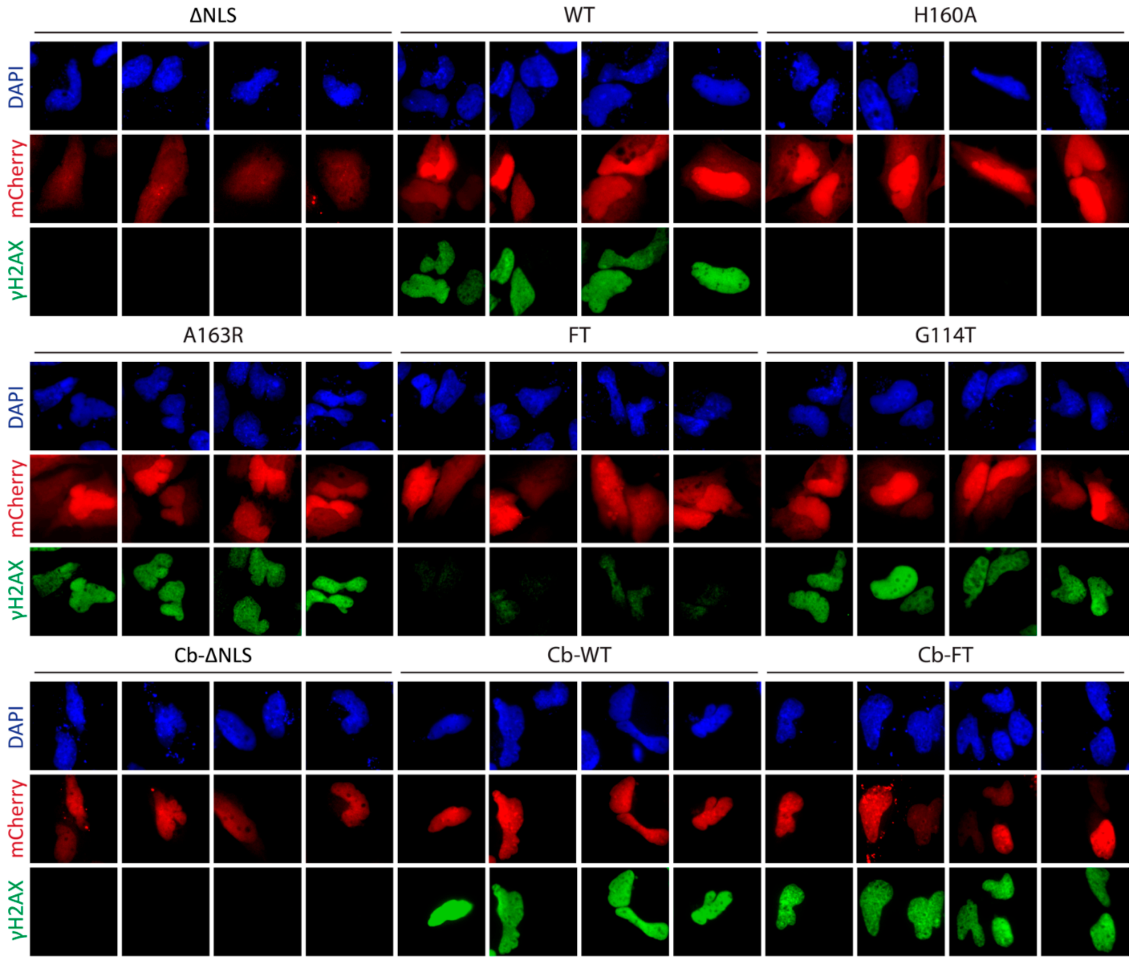

Figure S7. Representative images of cells from Fig 6. γH2AX immunofluorescence and mCherry fluorescence from HeLa cells transfected with pmCherry-C1-HducCdtB mutants with or without chromatibody (Cb) for 14h.

Table S1. Oligonucleotides used in this publication. Residues mutated for site-directed mutagenesis are represented in red. Primers used for pmCherry subcloning contain XhoI restriction site (in green) and EcoRI restriction site (in purple).

	Mutant	Forward Primer	Reverse Primer
Site-directed	**H160A**	TTTTACAGTGGCTGCTTTGGCCAC	AATACATCAGTACCAATGC
	**R117A**	GCAGTGAACTTAGCTATCGTGTCA	GTTTGCCCCAACATCTAAACGC
	**R144A**	CAATCTGCCCCTGCAGTAGGTATC	AGATTGAAGCACAGAAGAATCAGAA
	**N201A**	TGGTGATTTCGCTCGTGCGCCG	ACAACCATCCAGCTATATAC
	**G114T**	TTTAGATGTTACCGCAAACCGAGTGAAC	CGAGAATAATAAATATAGACCATATTTG
	**FT**	ATTTTTGCAGTGCATGCTTTGGCCACAG	TACATCAGTACCAATGCGGATACC
	**A163R**	CGCACAGGCGGTTCTGA	CAAAGCATGCACTGTAAAAAAT
	**ΔNLS**	CGCCGTCAAGCCGATGAA	AACATCTAAACGAGAATAATAAATATAGACCATATTTG
**pmC subcloning**		TATATACTCGAGCAAACTTGAGTGACTTCAAAGT	TATATAGAATTCTTAGCGATCACGAACAAAACT
